# Volatile chemical emissions from fragranced baby products

**DOI:** 10.1007/s11869-018-0593-1

**Published:** 2018-06-22

**Authors:** Neda Nematollahi, Augustine Doronila, Patrick J. Mornane, Alex Duan, Spas D. Kolev, Anne Steinemann

**Affiliations:** 1 Department of Infrastructure Engineering, Melbourne School of Engineering, The University of Melbourne, Melbourne, VIC 3010 Australia; 2 School of Chemistry, The University of Melbourne, Melbourne, VIC 3010 Australia; 3 College of Science and Engineering, James Cook University, Townsville, QLD 4811 Australia; 4 Climate, Atmospheric Sciences, and Physical Oceanography, Scripps Institution of Oceanography, University of California, San Diego, La Jolla, CA 92093 USA

**Keywords:** Baby products, Fragrance, Volatile organic compounds, Emissions, Ingredients

## Abstract

Fragranced consumer products have been associated with adverse effects on human health. Babies are exposed to a variety of fragranced consumer products, which can emit numerous volatile organic compounds (VOCs), some considered potentially hazardous. However, fragranced baby products are exempt from disclosure of all ingredients. Consequently, parents and the public have little information on product emissions. This study investigates VOCs emitted from a range of fragranced baby products, including baby hair shampoos, body washes, lotions, creams, ointments, oils, hair sprays, and fragrance. The products were analysed using gas chromatography/mass spectrometry (GC/MS) headspace analysis. Of the 42 baby products tested, 21 products made claims of green, organic, or all-natural. Results of the analysis found 684 VOCs emitted collectively from the 42 products, representing 228 different VOCs. Of these 684 VOCs, 207 are classified as potentially hazardous under federal regulations, representing 43 different VOCs. The most common VOCs emitted were limonene, acetaldehyde, ethanol, alpha-pinene, linalool, beta-myrcene, acetone, and beta-pinene. A comparison between ingredients emitted and ingredients listed reveals that only 5% of the 684 VOCs, including 12% of 207 potentially hazardous VOCs, were listed on the product label, safety data sheet, or website. More than 95% of both green and regular products emitted one or more potentially hazardous VOCs. Further, emissions of the most prevalent VOCs from green, organic, or all-natural products were not significantly different from regular products. Results from this study can help improve public awareness about emissions from baby products, with the aim to reduce pollutant exposure and potential adverse effects on babies.

**Electronic supplementary material:**

The online version of this article (10.1007/s11869-018-0593-1) contains supplementary material, which is available to authorized users.

## Introduction

Volatile organic compounds (VOCs) are a major category of pollutants, associated with adverse effects on human health (e.g., World Health Organization (WHO) 2018; National Institutes of Health (NIH) 2018; Safe Work Australia (SWA) 2018; Wallace 2001). Fragranced consumer products, widely used in society, emit numerous VOCs. Previous studies have found more than 150 different VOCs emitted from fragranced products, including terpenes such as limonene and alpha-pinene, and hazardous air pollutants such as formaldehyde and acetaldehyde (Steinemann 2015). Fragranced consumer products (or, for brevity, fragranced products) are defined as products with an added fragrance or scent, such as air fresheners, deodorizers, cleaning products, laundry supplies, household items, perfume, and personal care products (Steinemann 2015), as well as items for babies.

Exposure to fragranced consumer products is widespread. For instance, an estimated 98.5% of Australians are exposed to fragrance products at least once a week from their own use, others’ use, or both (Steinemann 2016, 2017). For babies, while comparable population studies are lacking in Australia, some international studies have found average use of baby shampoo, cleaning liquid or gel, and moisturising cream, respectively, as 2.9, 4.4, and 1.4 g/day (Gomez-Berrada et al. 2017) and 1.55, 2.91, and 2.99 g/day (Lee et al. 2017).

Exposure to fragranced consumer products has been associated with health problems such as asthma attacks, migraine headaches, respiratory difficulties, and allergic reactions among adults (Steinemann 2016, 2017; Weinberg et al. 2017). In young children, exposure to VOCs from household products, such as air fresheners, has been associated with infant diarrhoea and earache (Farrow et al. 2003). Babies can be exposed to product VOCs through epidermal, inhalation, and ingestion routes (Ott et al. 2007) as well as in utero (Bagasra et al. 2013).

The precise and full ingredients of fragranced products are not required to be fully disclosed (Lunny et al. 2017). The disclosure requirements depend on the type of product. For consumer products such as cleaning supplies, laundry products, air fresheners, and household items, no law in Australia requires disclosure of all ingredients. For personal care products, ingredients need to be listed on product labels, but the general term “fragrance” or “parfum” can be listed instead of the specific fragrance ingredients. However, a single “fragrance” in a product is typically a mixture of several dozen chemicals (Steinemann et al. 2011). Baby products are treated the same as adult products for the purposes of ingredient disclosure. Thus, fragranced baby consumer products (other than personal care products) have no requirement to disclose all ingredients, and baby personal care products are required to disclose ingredients except those in fragrance mixtures (Lunny et al. 2017).

Lack of knowledge of full ingredients may impair consumer choice. Consumers may be attracted to “natural” (or green or organic) products, assuming they may contain fewer chemicals of concern (Klaschka 2016). However, previous studies have discovered that products called natural, green, and organic emitted hazardous air pollutants similar to their regular counterparts (Steinemann 2015). Currently, there is no objective basis for claims of green or natural baby products. For organic baby products, Australian Certified Organic (ACO) provides a detailed standard (Australian certified Organic (ACO) 2018). Among the specifications, products should consist of at least 95% organic ingredients to be called “organic.” For products less than 95% organic, labels can make reference to the specific organic ingredients.

Previous research on VOC emissions from fragranced consumer products found the most common compounds were ethanol and limonene (Steinemann 2015), limonene (Dimitroulopoulou et al. 2015), and limonene and linalool (ter Burg et al. 2014). In addition, other terpenes such as alpha-pinene and beta-pinene and other volatiles such as acetone, acetaldehyde, benzyl acetate, and methanol were also common (Steinemann 2015). However, no prior research was identified that specifically analysed the VOCs emitted from fragranced baby products.

This article reports on the first known study to investigate the VOCs emitted from fragranced baby products, including both regular products and green products. In addition, this study compares the ingredients emitted from the products with the ingredients listed on the product label, safety data sheet, and website. Moreover, the emitted chemicals are compared with those classified as potentially hazardous under federal regulations. Finally, emissions between regular and green products are contrasted, as well as their links with hazard classifications and labelling.

## Materials and methods

Headspace GC/MS was used to identify chemicals emitted from 42 baby fragranced products in two categories: 21 regular and 21 green. The regular category includes 9 shampoos (hair shampoos, conditioners, and body washes), 9 lotions (lotions, creams, ointments, and oils), 2 hair sprays, and 1 fragrance spray. The green category includes 12 shampoos (hair shampoos, conditioners, and body washes), 8 lotions (lotions, creams, and oils), and 1 anti-bug spray. All of these 42 products were purchased from stores in Australia, including groceries, pharmacies, and organic food stores.

“Green” products are defined in this study as the products with the claim of “certified organic,” “certified green,” “green,” “organic,” “natural,” “no petrochemicals,” “non-toxic,” “plant-based,” and “essential oils” for the whole products or their ingredients on their product label, safety data sheet, or website. “Regular” products are the products not in the green category.

“Baby” products are defined in this study as products containing specific designations, such as wording (e.g., “baby shampoo”) or illustrations (e.g., picture of a baby), that they are intended for use on infants and toddlers.

“Fragranced” products are identified in this study as products containing fragrance, parfum, aromatic extracts, or essential oils.

Headspace GC/MS of the baby products was performed using a Shimadzu GC/MS-QP2010 Plus instrument coupled to an automated Shimadzu AOC-5000 sample injection system. Approximately 2 g of each baby product was weighed into a 10-mL amber vial. Each vial was then tightly sealed with a magnetic screw cap with a PTFE/silicone septum. Samples were incubated at 40 °C for 1 h immediately prior to injection of 2.5 mL of the headspace into the injection port heated at 240 °C (split ratio 25). Separation was performed on a BPX-VOL capillary column (30 m × 0.25 mm, 1.4 μm film thickness) using helium as the carrier gas (flow rate 30 cm/s). The oven temperature was kept at 35 °C for 3 min, then increased by 5 °C/min to 220 °C and held at this temperature for 5 min. The total run time was 45 min. The mass spectrometer ion source and interface temperatures were maintained at 200 and 240 °C, respectively. The mass spectrometer was operated in full scan mode between m/z 25 and 400. Blanks were analysed periodically each day to account for any background impurities. The volatile sample components were identified based on the mass spectral library of the National Institute of Standards and Technology NIST Version 2.0 (Stein 2008).

Two regulatory analyses were performed to identify whether chemicals emitted from baby products could be classified as (i) potentially hazardous under Australian regulations or (ii) potentially carcinogenic under the World Health Organization. For (i), Safe Work Australia (SWA) maintains a Hazardous Chemical Information System (HCIS) that permits identification of chemicals with hazard classifications (Safe Work Australia (SWA) 2018). For (ii), the World Health Organization, International Agency for Research on Cancer (IARC) (World Health Organization (WHO) 2018), evaluates the carcinogenic risk of chemicals to humans. However, these analyses do not imply that the chemicals identified are the only ones with potential hazards. Furthermore, these analyses did not assess whether the products that contained these chemicals could pose any potential hazards as a whole.

## Results and discussion

### VOCs emitted

A summary of all VOCs emitted from “green” and “regular” baby products is provided in Table 1. The term “VOC occurrences” refers to the number of individual VOC peaks detected by GC/MS, where each peak represents an ingredient in the product. The term “VOC identities” refers to the number of different VOCs, where each VOC is present in one or more of the products. Among the 42 products, a total of 684 VOC occurrences representing 228 VOC identities were detected. Each baby product emitted between 1 and 47 VOCs (see Supplementary Tables 1 and 2, available online). Complete data on VOCs identified for all 42 products, green products, and regular products are provided as Supplementary Tables 3, 4, and 5 (available online).

**Table 1:** VOCs emitted from 42 baby products compared with VOCs listed

Type	Number of products	Emitted		Listed	
		All VOCs	Potentially hazardous VOCs	All VOCs	Potentially hazardous VOCs
Regular	21	286 occurrences	95 occurrences	13 occurrences	9 occurrences
		137 identities	30 identities	5 identities	2 identities
Green	21	398 occurrences	112 occurrences	21 occurrences	16 occurrences
		154 identities	33 identities	4 identities	3 identities
Total	42	684 occurrences	207 occurrences	34 occurrences	25 occurrences
		228 identities	43 identities	7 identities	3 identities

### Most prevalent

Among the 42 products, the most prevalent VOCs (in at least 40% of the products) were limonene, acetaldehyde, ethanol, alpha-pinene, linalool, beta-myrcene, acetone, and beta-pinene. In regular products, the six most prevalent VOCs were ethanol, limonene, acetaldehyde, alpha-pinene, ethyl butyrate, and phenoxyethanol. In green products, the six most prevalent VOCs were limonene, linalool, acetaldehyde, beta-myrcene, alpha-pinene, and acetone. Among all the identified VOCs, limonene was the most common, found in 67% of the products (see Table 2 for the most prevalent compounds).

**Table 2:** Most prevalent compounds among baby products

Compound	CAS no.	Prevalence (no. of products)		
		Total	Regular	Green
All products (*n* = 42)				
Limonene*	138-86-3	28	11	17
Acetaldehyde*	75-07-0	23	10	13
Ethanol*	64-17-5	23	14	9
alpha-Pinene	80-56-8	21	8	13
Linalool	78-70-6	20	4	16
beta-Myrcene	123-35-3	19	6	13
Acetone*	67-64-1	17	5	12
beta-Pinene	127-91-3	17	7	10
Eucalyptol	470-82-6	14	4	10
Ethyl butyrate	105-54-4	13	8	5
3-Carene	13,466-78-9	12	3	9
Phenoxyethanol*	122-99-6	12	8	4
Benzyl alcohol*	100-51-6	11	4	7
Benzyl acetate	140-11-4	10	5	5
Camphene	79-92-5	10	3	7
Camphor	76-22-2	10	2	8
gamma-Terpinene	99-85-4	10	3	7
Regular products (*n* = 21)				
Ethanol*	64-17-5	14		
Limonene*	138-86-3	11		
Acetaldehyde*	75-07-0	10		
alpha-Pinene	80-56-8	8		
Ethyl butyrate	105-54-4	8		
Phenoxyethanol*	122-99-6	8		
Green products (*n* = 21)				
Limonene*	138-86-3	17		
Linalool	78-70-6	16		
Acetaldehyde*	75-07-0	13		
beta-Myrcene	123-35-3	13		
alpha-Pinene	80-56-8	13		
Acetone*	67-64-1	12		
Eucalyptol	470-82-6	10		
beta-Pinene	127-91-3	10		
Ethanol*	64-17-5	9		
3-Carene	13,466-78-9	9		
Linalool acetate	115-95-7	8		
Camphor	76-22-2	8		

*Classified as hazardous under Safe Work Australia, Hazardous Chemical Information System (SWA 2018)

### Regulatory classifications

Of the 684 VOC occurrences, 207 are classified as potentially hazardous under Australian regulations (Table 3), and 95% of products emitted at least one potentially hazardous VOC. Among the most prevalent VOCs (in at least 40% of the products), 50% are classified as potentially hazardous: limonene, acetaldehyde, ethanol, and acetone. Also, among these 684 VOC occurrences, 25 are classified as possibly carcinogenic to humans and 1 is classified as probably carcinogenic to humans (Table 4). However, none of these carcinogenic compounds were listed on any product label, safety data sheet, or website.

**Table 3:** Compounds classified as potentially hazardous* among baby products

Compound	CAS no.	Prevalence (no. of products)		
		Total	Regular	Green
Limonene	138-86-3	28	11	17
Acetaldehyde	75-07-0	23	10	13
Ethanol	64-17-5	23	14	9
Acetone	67-64-1	17	5	12
Phenoxyethanol	122-99-6	12	8	4
Benzyl alcohol	100-51-6	11	4	7
Isopropyl alcohol	67-63-0	9	4	5
Ethyl Acetate	141-78-6	8	5	3
1-Octanol	111-87-5	7	1	6
Pentane	109-66-0	6	3	3
Isoamyl acetate	123-92-2	5	4	1
2,4-Dimethylhexane	589-43-5	4	0	4
Toluene	108-88-3	4	1	3
2-Methylbutyl acetate	624-41-9	3	2	1
Cyclohexane	110-82-7	3	1	2
Hexane	110-54-3	3	1	2
Methanol	67-56-1	3	2	1
Octamethylcyclotetrasiloxane	556-67-2	3	3	0
Propanal	123-38-6	3	3	0
2-Butenal	4170-30-3	2	1	1
2-Methyl-1-propene	115-11-7	2	0	2
2-Methylpentane	107-83-5	2	0	2
3-Methylhexane	589-34-4	2	1	1
Benzaldehyde	100-52-7	2	1	1
Butyl acetate	123-86-4	2	1	1
Isobutyl 2-methyl-2-propenoate	97-86-9	2	0	2
Tetrahydrofuran	109-99-9	2	2	0
(2Z)-3,7-dimethylocta-2,6-dienal	106-26-3	1	1	0
1-Hexanol	111-27-3	1	1	0
2,4-Dimethylpentane	108-08-7	1	0	1
2,4-Pentanedione	123-54-6	1	0	1
3-Methylpentane	96-14-0	1	0	1
Amyl acetate	628-63-7	1	0	1
Benzyl benzoate	120-51-4	1	1	0
Butane	106–97-8	1	1	0
Citral	5392-40-5	1	0	1
Dichloromethane	75-09-2	1	1	0
Ethyl lactate	97-64-3	1	0	1
Ethyl methyl ether	540-67-0	1	1	0
Ethyl propionate	105–37-3	1	1	0
Methylcyclohexane	108-87-2	1	0	1
Octane	111-65-9	1	0	1
Pentyl acetate	628-63-7	1	0	1

*Classified as hazardous under Safe Work Australia, Hazardous Chemical Information System (SWA 2018)

**Table 4:** Compounds classified for carcinogenic risk

Compound	CAS no.	Classification group	Prevalence (no. of products)		
			Total	Regular	Green
Acetaldehyde	75-07-0	2B	23	10	13
Isopropyl alcohol	67-63-0	3	9	4	5
Toluene	108-88-3	3	4	1	3
Tetrahydrofuran	109-99-9	2B	2	2	0
2-Butenal	4170-30-3	3	2	1	1
Dichloromethane	75-09-2	2A	1	1	0

Group 2A: Probably carcinogenic to humans, Group 2B: Possibly carcinogenic to humans, Group 3: Not classifiable as to its carcinogenicity to humans (World Health Organization (WHO) 2018)

### Green products

Among the green products, 20 emitted at least 1 VOCs classified as potentially hazardous (Supplementary Table 1, available online). Among the regular products, 20 emitted at least 1 VOCs classified as potentially hazardous (Supplementary Table 2, available online). Among the most prevalent VOCs also classified as potentially hazardous, 75% are similar among regular and green products. Comparing the 21 regular and 21 green products, no significant difference was found in the most prevalent VOCs (*p* < 0.05, *t* test).

### Listing of ingredients

Among all the 684 VOC occurrences, only 34 were listed on any baby product label, safety data sheet, or website. Thus, 5% of all volatile chemicals identified were listed. Further, among all the 207 VOCs classified as potentially hazardous, only 25 were listed on any baby product label, safety data sheet, or website. Thus, 88% of all potentially hazardous volatile chemicals identified were undisclosed.

### Limitations

This analysis focused on the primary VOC emissions, even though secondary pollutants (such as formaldehyde) could also be generated. Further, the study focused on VOCs, even though other chemical classes (such as semi-volatile or non-volatile organic compounds) could also pose potential hazards. Finally, the relative safety or hazard of products depends on many factors (e.g., VOC concentrations, frequency, and duration of exposure), and this study focused exclusively on identifying VOC emissions.

## Conclusions

This study provides the results of VOCs emitted from 42 common fragranced baby products, finding a total of 684 VOCs emitted (representing 228 different VOCs), with 207 VOCs emitted (representing 43 different VOCs) classified as potentially hazardous. However, only 5% of all VOCs and 12% of potentially hazardous VOCs were listed on any product label, safety data sheet, or website. Moreover, emissions of the most prevalent VOCs from green, organic, and natural fragranced baby products were not significantly different from regular baby products. The results of this study can contribute to improved awareness of product ingredients and to reduce the risk of exposure for babies.

## Electronic supplementary material

ESM 1 (DOC 366 kb)

ESM 2 (DOC 284 kb)

ESM 3 (DOC 21 kb)

ESM 4 (DOC 19 kb)

ESM 5 (DOC 18 kb)
