# Supplementary material for: Volatile chemical emissions from fragranced baby products
Source: Air Qual Atmos Health. 2018 Jun 22;11(7):785–90. doi: 10.1007/s11869-018-0593-1 (PMC6097056; doi:10.1007/s11869-018-0593-1)
Supplement: Supplementary file 4 — (DOC 19 kb) [file 11869_2018_593_MOESM4_ESM.doc]

**Supplementary Table 4**

**Green Baby Products (n=21):**

| Compound | CAS # | Prevalence (# of products) |
| --- | --- | --- |
| Limonene* | 138-86-3 | 17 |
| Linalool | 78-70-6 | 16 |
| Acetaldehyde* | 75-07-0 | 13 |
| beta-Myrcene | 123-35-3 | 13 |
| alpha-Pinene | 80-56-8 | 13 |
| Acetone* | 67-64-1 | 12 |
| Eucalyptol | 470-82-6 | 10 |
| beta-Pinene | 127-91-3 | 10 |
| Ethanol* | 64-17-5 | 9 |
| 3-Carene | 13466-78-9 | 9 |
| Linalool acetate | 115-95-7 | 8 |
| Camphor | 76-22-2 | 8 |
| Benzyl alcohol* | 100-51-6 | 7 |
| Camphene | 79-92-5 | 7 |
| gamma-Terpinene | 99-85-4 | 7 |
| 1-Octanol* | 111-87-5 | 6 |
| beta-Phellandrene | 555-10-2 | 6 |
| alpha-Phellandrene | 99-83-2 | 6 |
| Isopropyl alcohol* | 67-63-0 | 5 |
| Benzyl acetate | 140-11-4 | 5 |
| Ethyl butyrate | 105-54-4 | 5 |
| beta-trans-Ocimene | 3779-61-1 | 5 |
| Hexyl acetate | 142-92-7 | 5 |
| Neryl acetate | 141-12-8 | 5 |
| Phenoxyethanol* | 122-99-6 | 4 |
| Hexanal | 66-25-1 | 4 |
| alpha-Terpineol | 98-55-5 | 4 |
| 2,4-Dimethylhexane* | 589-43-5 | 4 |
| Terpinolene | 586-62-9 | 4 |
| Isobutyl isobutyrate | 97-85-8 | 4 |
| Ocimene | 13877-91-3 | 4 |
| Ethyl acetate* | 141-78-6 | 3 |
| Ethyl 2-methylbutyrate | 7452-79-1 | 3 |
| Pentane* | 109-66-0 | 3 |
| 3-Octanone | 106-68-3 | 3 |
| 2-Methylfuran | 534-22-5 | 3 |
| Toluene* | 108-88-3 | 3 |
| Cyclohexane* | 110-82-7 | 2 |
| 1-Undecanol | 112-42-5 | 2 |
| Undecane | 1120-21-4 | 2 |
| Phenylethyl alcohol | 60-12-8 | 2 |
| Dihydromyrcenol | 18479-58-8 | 2 |
| Hexane* | 110-54-3 | 2 |
| Sabinene | 3387-41-5 | 2 |
| Tridecane | 629-50-5 | 2 |
| Ethyl linalool | 10339-55-6 | 2 |
| Dodecane | 112-40-3 | 2 |
| 2-Methylpentane* | 107-83-5 | 2 |
| 2-Methyl-1-propene* | 115-11-7 | 2 |
| 2-tert-Butylcyclohexanol | 13491-79-7 | 2 |
| Geraniol acetate | 105-87-3 | 2 |
| 3,7-Dimethyl-3-octanol | 78-69-3 | 2 |
| 1,1-Dimethylallyl alcohol | 115-18-4 | 2 |
| Methyl hexyl ether | 4747-07-3 | 2 |
| Isobutyl 2-methyl-2-propenoate* | 97-86-9 | 2 |
| Ethyl 2-methylcyclopropanecarboxylate | 20913-25-1 | 2 |
| 1-Octen-3-yl-acetate | 2442-10-6 | 2 |
| Hexyl butyrate | 2639-63-6 | 2 |
| Octyl butyrate | 110-39-4 | 2 |
| 4-Terpineol | 562-74-3 | 2 |
| Methylcyclopentane | 96-37-7 | 2 |
| Amyl senecioate | 56922-72-6 | 2 |
| Ocimenol | 5986-38-9 | 2 |
| β-Citronellol | 106-22-9 | 2 |
| m-Cymene | 535-77-3 | 2 |
| cis-3-Hexenol | 928-96-1 | 1 |
| Carvone | 99-49-0 | 1 |
| Piperonal | 120-57-0 | 1 |
| Cyclododecane | 294-62-2 | 1 |
| trans-beta-Ionone | 79-77-6 | 1 |
| 4-tert-Butylcyclohexyl acetate | 32210-23-4 | 1 |
| Pentanal | 110-62-3 | 1 |
| 3-Octanol | 589-98-0 | 1 |
| 2,4-Dimethylheptane | 2213-23-2 | 1 |
| Methanol* | 67-56-1 | 1 |
| alpha-Thujene | 2867-05-2 | 1 |
| Bromocyclohexane | 108-85-0 | 1 |
| Isoamyl acetate* | 123-92-2 | 1 |
| 2-Butenal* | 4170-30-3 | 1 |
| 2-Methylenehexanal | 1070-66-2 | 1 |
| Methyl octanoate | 111-11-5 | 1 |
| 2-Methylbutyl acetate* | 624-41-9 | 1 |
| Amyl acetate* | 628-63-7 | 1 |
| 1-Dodecanol | 112-53-8 | 1 |
| 2,3,4-Trimethylhexane | 921-47-1 | 1 |
| Ethyl 2-methylpentanoate | 39255-32-8 | 1 |
| Methyl phenylcarbinyl acetate | 93-92-5 | 1 |
| 3,7-Dimethyloctan-3-yl acetate | 20780-48-7 | 1 |
| 3-Methyl-1-octene | 13151-08-1 | 1 |
| 1-Decene | 872-05-9 | 1 |
| Prenyl acetate | 1191-16-8 | 1 |
| 4-Hexen-1-ol, acetate | 72237-36-6 | 1 |
| 1-Methyl-3-cyclohexene-1-carbaldehyde | 931-96-4 | 1 |
| Allyl heptanoate | 142-19-8 | 1 |
| 2-Phenoxyethyl isobutyrate | 103-60-6 | 1 |
| Benzaldehyde* | 100-52-7 | 1 |
| 3-Methylpentane* | 96-14-0 | 1 |
| 2,2-Dimethyldecane | 17302-37-3 | 1 |
| 2-Phenoxyethyl isobutyrate | 103-60-6 | 1 |
| 2-Methyl-1-butene | 563-46-2 | 1 |
| 3-Methyl-2-butenoic acid, octyl ester | 56500-47-1 | 1 |
| Octanal | 124-13-0 | 1 |
| 2,2,6,6-Tetramethylcyclohexanol | 6948-41-0 | 1 |
| Borneol | 10385-78-1 | 1 |
| Dihydrocarveol | 38049-26-2 | 1 |
| 1,1-Dimethyl-2-(2-methyl-1-propenyl)cyclopropane | 33422-32-1 | 1 |
| 2,4-Dimethylpentane* | 108-08-7 | 1 |
| Octane* | 111-65-9 | 1 |
| 2,4-Pentanedione* | 123-54-6 | 1 |
| Methylcyclohexane* | 108-87-2 | 1 |
| 3,5,5-Trimethylhexyl acetate | 58430-94-7 | 1 |
| Linalyl anthranilate | 7149-26-0 | 1 |
| 2-Carene | 554-61-0 | 1 |
| 3-Methylhexane* | 589-34-4 | 1 |
| Decamethylcyclopentasiloxane | 541-02-6 | 1 |
| 3-Methylfuran | 930-27-8 | 1 |
| Butyl acetate* | 123-86-4 | 1 |
| (Z)-beta-ocimene | 3338-55-4 | 1 |
| 2,6-Octadiene, 2,4-dimethyl- | 63843-03-8 | 1 |
| 3-Octyl acetate | 4864-61-3 | 1 |
| 4,4,6,6-Tetramethylbicyclo[3.1.0]hex-2-ene | 19487-09-3 | 1 |
| 2-Methyl pentyl isobutyrate | 84254-82-0 | 1 |
| Lavandulyl acetate | 25905-14-0 | 1 |
| (Z)-beta-farnesene | 28973-97-9 | 1 |
| Bicyclo[5.2.0]nonane, 4-ethenyl-4,8,8-trimethyl-2-methylene- | 242794-76-9 | 1 |
| Ethyl lactate* | 97-64-3 | 1 |
| Ethyl isovalerate | 108-64-5 | 1 |
| D,l-isobornyl acetate | 92618-89-8 | 1 |
| 1,4-Pentadiene | 591-93-5 | 1 |
| 2-Methyl-2-butene | 513-35-9 | 1 |
| Dimethyl sulfide | 75-18-3 | 1 |
| Isobutyraldehyde | 78-84-2 | 1 |
| 3-Methyl-1-cyclopentene | 1120-62-3 | 1 |
| Isovaleraldehyde | 590-86-3 | 1 |
| 2-Methylbutyraldehyde | 96-17-3 | 1 |
| 2-Ethylfuran | 3208-16-0 | 1 |
| 3,3-Dimethylallyl bromide | 870-63-3 | 1 |
| 2,3-Dehydro-1,8-cineole | 92760-25-3 | 1 |
| 6-Methyl-5-hepten-2-one | 110-93-0 | 1 |
| 4-Carene | 29050-33-7 | 1 |
| 4-Nonanone | 4485-09-0 | 1 |
| Acetylmethylcyclohexene,4-acetyl-1-methyl-1-cyclohexene | 70286-20-3 | 1 |
| (R)-(+)-citronellal | 2385-77-5 | 1 |
| 4,5,6,7-Tetrahydro-3,6-dimethylbenzofuran | 494-90-6 | 1 |
| (1R,4S)-p-menthan-3-one | 14073-97-3 | 1 |
| L-menthol | 2216-51-5 | 1 |
| Methyl salicylate | 119-36-8 | 1 |
| cis-3,7-Dimethyl-2,6-octadien-1-ol | 106-25-2 | 1 |
| (2Z)-3,7-dimethylocta-2,6-dienal* | 106-26-3 | 1 |
| Dl-menthyl acetate | 16409-45-3 | 1 |
| Citral* | 5392-40-5 | 1 |
| β-Caryophyllene | 87-44-5 | 1 |
| alpha-Himachalen | 3853-83-6 | 1 |
| alpha-Longipinene | 5989-08-2 | 1 |

*Classified as hazardous under Safe Work Australia, Hazardous Chemical Information System (SWA 2018)
