# Supplementary material for: Volatile chemical emissions from fragranced baby products
Source: Air Qual Atmos Health. 2018 Jun 22;11(7):785–90. doi: 10.1007/s11869-018-0593-1 (PMC6097056; doi:10.1007/s11869-018-0593-1)
Supplement: Supplementary file 5 — (DOC 18 kb) [file 11869_2018_593_MOESM5_ESM.doc]

**Supplementary Table 5**

**Regular Baby Products (n=21):**

| Compound | CAS # | Prevalence (# of products) |
| --- | --- | --- |
| Ethanol* | 64-17-5 | 14 |
| Limonene* | 138-86-3 | 11 |
| Acetaldehyde* | 75-07-0 | 10 |
| alpha-Pinene | 80-56-8 | 8 |
| Ethyl butyrate | 105-54-4 | 8 |
| Phenoxyethanol* | 122-99-6 | 8 |
| beta-Pinene | 127-91-3 | 7 |
| beta-Myrcene | 123-35-3 | 6 |
| Ethyl 2-methylbutyrate | 7452-79-1 | 6 |
| Acetone* | 67-64-1 | 5 |
| Benzyl acetate | 140-11-4 | 5 |
| Ethyl acetate* | 141-78-6 | 5 |
| Linalool | 78-70-6 | 4 |
| Eucalyptol | 470-82-6 | 4 |
| Benzyl alcohol* | 100-51-6 | 4 |
| Isopropyl alcohol* | 67-63-0 | 4 |
| Phenylethyl alcohol | 60-12-8 | 4 |
| cis-3-Hexenol | 928-96-1 | 4 |
| 4-tert-Butylcyclohexyl acetate | 32210-23-4 | 4 |
| Isoamyl acetate* | 123-92-2 | 4 |
| Nonadecane | 629-92-5 | 4 |
| 3-Carene | 13466-78-9 | 3 |
| Camphene | 79-92-5 | 3 |
| beta-Phellandrene | 555-10-2 | 3 |
| gamma-Terpinene | 99-85-4 | 3 |
| Pentane* | 109-66-0 | 3 |
| Cyclododecane | 294-62-2 | 3 |
| Octamethylcyclotetrasiloxane* | 556-67-2 | 3 |
| Propanal* | 123-38-6 | 3 |
| (Z)-7-tetradecene | 41446-60-0 | 3 |
| Isoamyl butylate | 106-27-4 | 3 |
| Terpinolene | 586-62-9 | 2 |
| 4-Carene | 29050-33-7 | 2 |
| Camphor | 76-22-2 | 2 |
| Hexyl acetate | 142-92-7 | 2 |
| alpha-Phellandrene | 99-83-2 | 2 |
| Undecane | 1120-21-4 | 2 |
| Dihydromyrcenol | 18479-58-8 | 2 |
| alpha-Isomethyl ionone | 127-51-5 | 2 |
| 2-tert-Butylcyclohexanol | 13491-79-7 | 2 |
| Methanol* | 67-56-1 | 2 |
| alpha-Thujene | 2867-05-2 | 2 |
| 1,3,4-Trimethyl-3-cyclohexen-1-carboxaldehyde | 40702-26-9 | 2 |
| 1-Tridecene | 2437-56-1 | 2 |
| Tris(trimethylsilyl) borate | 4325-85-3 | 2 |
| Decamethylcyclopentasiloxane | 541-02-6 | 2 |
| Tetrahydrofuran* | 109-99-9 | 2 |
| m-Cymene | 535-77-3 | 2 |
| Tetrahydrolinalool | 57706-88-4 | 2 |
| Amyl butyrate | 540-18-1 | 2 |
| Ethyl hexanoate | 123-66-0 | 2 |
| 2-Methylbutyl acetate* | 624-41-9 | 2 |
| Linalool acetate | 115-95-7 | 1 |
| 1-Octanol* | 111-87-5 | 1 |
| beta-trans-Ocimene | 3779-61-1 | 1 |
| alpha-Terpineol | 98-55-5 | 1 |
| Cyclohexane* | 110-82-7 | 1 |
| Hexane* | 110-54-3 | 1 |
| Piperonal | 120-57-0 | 1 |
| Tridecane | 629-50-5 | 1 |
| Dodecane | 112-40-3 | 1 |
| 1-Hexanol* | 111-27-3 | 1 |
| trans-beta-Ionone | 79-77-6 | 1 |
| Toluene* | 108-88-3 | 1 |
| Dichloromethane* | 75-09-2 | 1 |
| Hexamethylcyclotrisiloxane | 541-05-9 | 1 |
| 2,6-Dimethyl-2-heptanol | 13254-34-7 | 1 |
| 2-Butene* | 107-01-7 | 1 |
| 2-Methyl-1-pentene | 763-29-1 | 1 |
| 2,7-Dimethyl-1,7-octadiene | 59840-10-7 | 1 |
| 3,7-Dimethyl-1,6-octadiene | 10281-56-8 | 1 |
| 1,4-Hexadiene | 592-45-0 | 1 |
| cis-6-Nonenyl acetate | 76238-22-7 | 1 |
| 4-tert-Pentylcyclohexanol | 20698-30-0 | 1 |
| Nopyl acetate | 128-51-8 | 1 |
| Isobutyl cyanate | 1768-25-8 | 1 |
| 3-Methylhexane* | 589-34-4 | 1 |
| 3,4-Dimethylheptane | 922-28-1 | 1 |
| Ethyl methyl ether* | 540-67-0 | 1 |
| Methacrolein | 78-85-3 | 1 |
| Trimethylsilanol | 1066-40-6 | 1 |
| 1,1'-Oxydi-2-propanol | 110-98-5 | 1 |
| 2-(2-Hydroxypropoxy)-1-propanol | 106-62-7 | 1 |
| 2,2'-Oxydipropanol | 108-61-2 | 1 |
| 7-Hydroxycitronellal | 107-75-5 | 1 |
| 1,5-Hexadien-3-ol | 924-41-4 | 1 |
| (+)-Camphene | 5794-03-6 | 1 |
| Isoborneol | 124-76-5 | 1 |
| 2,9-Dimethyl-5-decyne | 19550-56-2 | 1 |
| Pentylidenecyclopentane | 53366-55-5 | 1 |
| (E)-3,3-Dimethylcyclohexylideneacetaldehyde | 26532-25-2 | 1 |
| Dl-menthol | 15356-70-4 | 1 |
| Benzaldehyde* | 100-52-7 | 1 |
| Ethyl benzoate | 93-89-0 | 1 |
| Butyl acetate* | 123-86-4 | 1 |
| cis-1,3,5-Trimethylcyclohexane | 1795-27-3 | 1 |
| 1-Ethyl-2-methylcyclohexane | 3728-54-9 | 1 |
| 2,6-Dimethyl octane | 2051-30-1 | 1 |
| 2-Butyl-1-octanol | 3913-02-8 | 1 |
| 1-Butyl-2-propylcyclopentane | 62199-50-2 | 1 |
| 3-Methylnonane | 5911-04-6 | 1 |
| 1-Ethyl-2-propylcyclohexane | 62238-33-9 | 1 |
| 1,2-Diethylcyclohexane | 824-43-1 | 1 |
| 1-Methyl-2-propylcyclohexan | 4291-79-6 | 1 |
| 2,5,5-Trimethylheptane | 1189-99-7 | 1 |
| Butylcyclohexane | 1678-93-9 | 1 |
| trans-Decahydronaphthalene | 493-02-7 | 1 |
| Ethyl propionate* | 105-37-3 | 1 |
| Ethyl 2-methylpentanoate | 39255-32-8 | 1 |
| Ethyl nonanoate | 123-29-5 | 1 |
| Pentyl acetate* | 628-63-7 | 1 |
| Dipentyl ether | 693-65-2 | 1 |
| 2-Methylbutyl 2-methylbutyrate | 2445-78-5 | 1 |
| Isoamyl isovalerate | 659-70-1 | 1 |
| 2-Methylbutyl isovalerate | 2445-77-4 | 1 |
| Benzyl benzoate* | 120-51-4 | 1 |
| Ethyl isovalerate | 108-64-5 | 1 |
| 2,2-Dimethyldecane | 17302-37-3 | 1 |
| 3,5,5-Trimethylhexyl acetate | 58430-94-7 | 1 |
| 2,6-Dichlorobenzyl alcohol | 15258-73-8 | 1 |
| 2,6-Di-tert-butyl-4-methylphenol | 128-37-0 | 1 |
| Methyl isopropyl ether | 598-53-8 | 1 |
| 2-Ethyl-4-methyl-1,3-dioxolane | 4359-46-0 | 1 |
| 1,3-Dioxane | 505-22-6 | 1 |
| cis-3-Hexenyl acetate | 3681-71-8 | 1 |
| 2,4,6-Trimethyl-3-cyclohexene-1-carboxaldehyde | 1423-46-7 | 1 |
| Allyl heptanoate | 142-19-8 | 1 |
| (Z+E)-2-methyl-2-(4-methyl-3-pentenyl) cyclopropane carbaldehyde | 97231-35-1 | 1 |
| p-Anisaldehyde | 123-11-5 | 1 |
| Butane* | 106-97-8 | 1 |
| Isobutyraldehyde | 78-84-2 | 1 |
| Isovaleraldehyde | 590-86-3 | 1 |
| 2-Methylbutyraldehyde | 96-17-3 | 1 |
| 2-Pentanone | 107-87-9 | 1 |
| o-Cymene | 527-84-4 | 1 |
| (Z)-sabinene hydrate | 15537-55-0 | 1 |
| (−)-Terpinen-4-ol | 20126-76-5 | 1 |

*Classified as hazardous under Safe Work Australia, Hazardous Chemical Information System (SWA 2018)
